# Bridging population and cell: modelling complex diseases with human induced pluripotent stem cells

**DOI:** 10.1038/s41431-026-02071-4

**Published:** 2026-03-31

**Authors:** Eva S. van Zanten, Elizabeth A. Loehrer, Joyce B. J. van Meurs, Roberto Narcisi, Joost H. Gribnau, Raymond A. Poot, Hieab H. H. Adams

**Affiliations:** 1 Department of Clinical Genetics, Radboud UMC, Nijmegen, The Netherlands; 2 Department of Clinical Genetics, Erasmus MC, Rotterdam, The Netherlands; 3 Department of Internal Medicine, Erasmus MC, Rotterdam, The Netherlands; 4 Department of Orthopaedics & Sports Medicine, Erasmus MC, Rotterdam, The Netherlands; 5 Department of Developmental Biology, Erasmus MC, Rotterdam, The Netherlands; 6 Latin American Brain Health (BrainLat), Universidad Adolfo Ibáñez, Santiago, Chile

**Keywords:** Induced pluripotent stem cells, Complex diseases, Functional genomics, Genetic variation, Disease modelling, Functional genomics, Disease model, Induced pluripotent stem cells

## Abstract

Induced pluripotent stem cells (iPSCs) have emerged as a powerful tool in biomedical research, enabling the study of cellular function and early disease mechanisms within patient-specific genetic contexts. Traditionally, iPSCs have been used to model monogenic diseases, where highly penetrant variants produce robust cellular phenotypes detectable in few cell lines. Recent advances in scalability and standardisation now enable systematic comparisons across many donors. This development is particularly relevant for complex diseases, which are driven by numerous genetic variants with small individual effects and therefore require population-scale designs to resolve genotype–phenotype relationships. However, several limitations of iPSC technology continue to challenge the reliability and reproducibility of such studies, constraining their translational relevance. Here, we review the challenges and opportunities of using iPSCs to model complex diseases, structured around three key themes: detecting subtle effects, modelling environmental context, and expanding genetic diversity.

## Introduction

Complex diseases arise from interactions between genetic variation and environmental factors and include a range of common diseases, such as cardiovascular, neurological, and musculoskeletal diseases, that together constitute a major global health burden. Many of these conditions are age-related, with cellular pathology emerging long before clinical symptoms manifest. Capturing such early disease mechanisms requires human model systems that can integrate genetic variation, cellular complexity, and environmental factors.

Genome-wide association studies (GWAS) have identified numerous genetic variants associated with complex diseases [1, 2], highlighting the significant contribution of genetic factors to complex disease phenotypes. However, translating these genetic associations into mechanistic insights remains a central challenge, since GWAS signals alone rarely resolve how genetic risk manifests in specific cellular contexts, necessitating complementary functional model systems to bridge association and mechanism [3]. Traditionally, modelling systems have provided valuable snapshots of advanced disease stages, but fail to fully capture the tissue-specific, developmental, and environmental contexts in which pathogenic mechanisms occur.

Induced pluripotent stem cells (iPSCs), somatic cells reprogrammed into a pluripotent state [4], offer a unique opportunity to overcome several of these limitations. iPSCs can be differentiated into various cell types while retaining the genetic background of the donor [5], thereby enabling systematic studies of the contribution of genetic variation to disease-relevant cellular phenotypes. Moreover, the process of iPSC differentiation enables researchers to follow cellular trajectories from pluripotency towards mature phenotypes, providing a window into pathogenic mechanisms that precede clinical symptoms.

Despite these advantages, several technical and biological factors, such as induced variability and limited genetic diversity, may constrain the fidelity of iPSC-based complex disease models. Continued methodological refinement will therefore be essential to improve their robustness and translational potential.

This review focuses on three foundational aspects that are critical for advancing iPSC-based modelling of complex diseases: large-scale and standardised iPSC derivation, incorporation of environmental factors, and representation of genetic diversity. We discuss how progress across these domains can transform the iPSC technology into a versatile platform to bridge the gap between genetic risk and cellular mechanisms of disease.

## Challenges and opportunities in iPSC-based disease modelling

Genome-wide association studies (GWAS) have identified hundreds of thousands of variants associated with complex traits and diseases [6], yet translating these associations into causal cellular mechanisms remains a major challenge. Resources such as the Genotype-Tissue Expression (GTEx) consortium [7] have provided comprehensive maps of genetic regulation across post-mortem tissues, but such static tissue snapshots cannot capture dynamic gene regulation, cellular heterogeneity, or developmental transitions. A cellular system that recapitulates human physiology is therefore required to interpret complex disease risk at a functional level.

**Fig. 1:**
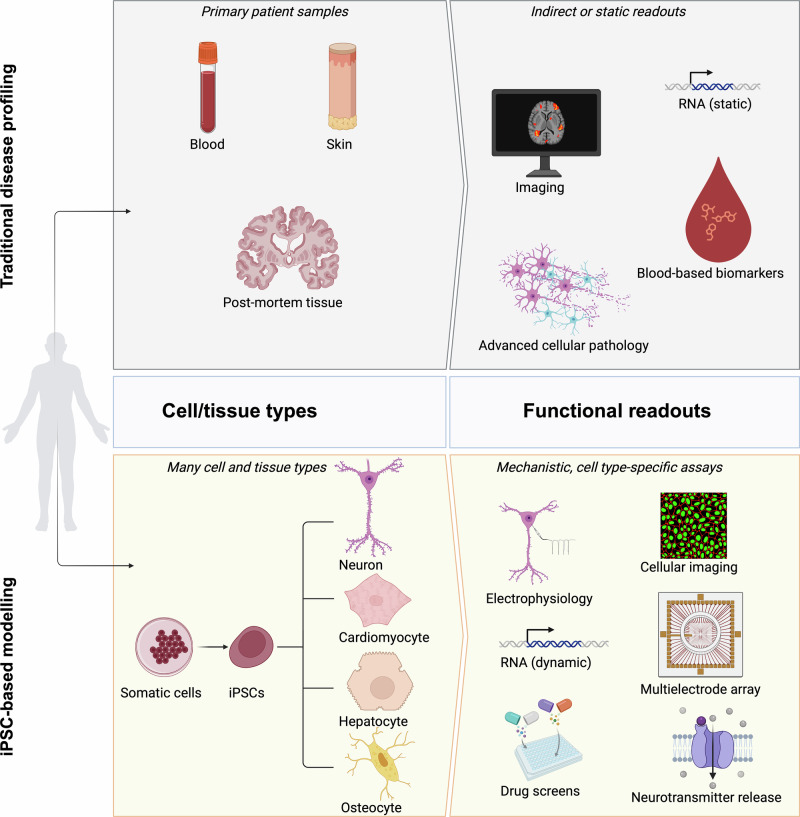
Advantages of iPSCs as compared to traditional modelling systems in modelling complex diseases. Traditional modelling systems are restricted to peripheral or post-mortem tissues, which have limited translational relevance for complex diseases (upper panel). iPSCs enable access to otherwise inaccessible cell types and multicellular systems derived from easily obtainable tissues (lower panel) and can therefore yield many more specific insights into complex disease mechanisms. iPSC induced pluripotent stem cell. Created with BioRender.

iPSC models can address some of these limitations by enabling experimental access to genetically defined human cell types. iPSC lines can be generated from large cohorts of genetically diverse individuals, enabling association analyses between genetic variation and cellular phenotypes in defined human cell types. These cellular traits can be chosen to reflect molecular or functional processes relevant to the clinical phenotypes identified by GWAS, thereby providing an experimentally accessible intermediate between genotype and disease. Moreover, iPSCs can be differentiated into otherwise inaccessible lineages, such as neurons, cardiomyocytes, and hepatocytes, thereby providing a system to connect genetic variation to molecular and cellular phenotypes (Fig. 1). iPSCs can be used to generate monocultures for high-throughput perturbations, co-cultures that capture intercellular communication, or three-dimensional organoids that can recapitulate aspects of tissue architecture and physiology. For instance, iPSC-derived neuronal cells can self-organise into cerebral organoids expressing brain region and layer-specific markers [8]. Organoids derived from Alzheimer’s disease patients recapitulate hallmark features of the disease, including neuronal hyperexcitability, Aβ aggregation, and tau hyperphosphorylation [9, 10], illustrating the capacity of iPSC-derived systems to capture disease-relevant mechanisms. However, studies comparing primary and iPSC-derived organoids have shown that brain organoids predominantly reflect mid-gestation foetal stages rather than adult tissue, with limited synaptic and metabolic maturity [11, 12], constraining modelling late-onset diseases. Advances such as vascularization [13, 14], immune cell co-cultures [13], and microfluidic perfusion [15] have increased the physiological fidelity of iPSC-derived brain organoids, revealing more complex disease phenotypes. Yet even these models remain developmentally young, underscoring the need for approaches that better represent the biological context of ageing-related disease mechanisms.

Another important advantage of iPSCs is their capacity to recapitulate key developmental milestones such as primitive streak formation, germ cell specification, and somitogenesis [16–20]. This enables researchers to examine how genetic variation shapes fundamental cellular programmes that influence disease susceptibility, even when clinical pathology arises later in life. For example, iPSC-based models of schizophrenia have revealed early neurodevelopmental abnormalities, including imbalanced excitatory and inhibitory neuronal differentiation, altered progenitor proliferation, and cortical layer disorganization [21], illustrating how disrupted developmental trajectories can contribute to disease risk.

## Navigating core challenges in iPSC-based complex disease modelling

Nearly two decades of iPSC-based modelling have yielded important insights into both the strengths and limitations of this system. While iPSCs have shown considerable promise for disease modelling, drug screening, and regenerative medicine, several fundamental challenges remain that require careful experimental consideration (reviewed in refs. [22–25]).

Some of these limitations stem from the artificial context in which iPSCs are generated. Every technical step that drives somatic cells to pluripotency can introduce variability at genetic [26], epigenetic [27], transcriptomic [28], and proteomic [29] levels, particularly when performed manually. This may cause iPSC-derived cells to deviate from the primary cell types they are intended to model. Across differentiations, iPSC-derived cells generally recapitulate the broad lineage identity of primary cells but often represent immature states and display heterogeneity in subtype composition and functional maturity [30, 31]. Ongoing efforts towards protocol standardisation [32], automated cell culturing [33, 34], and benchmarking against single-cell reference atlases [35], can greatly improve the reproducibility and validity of iPSC-based models, paving the way for a next generation of iPSC research.

**Fig. 2:**
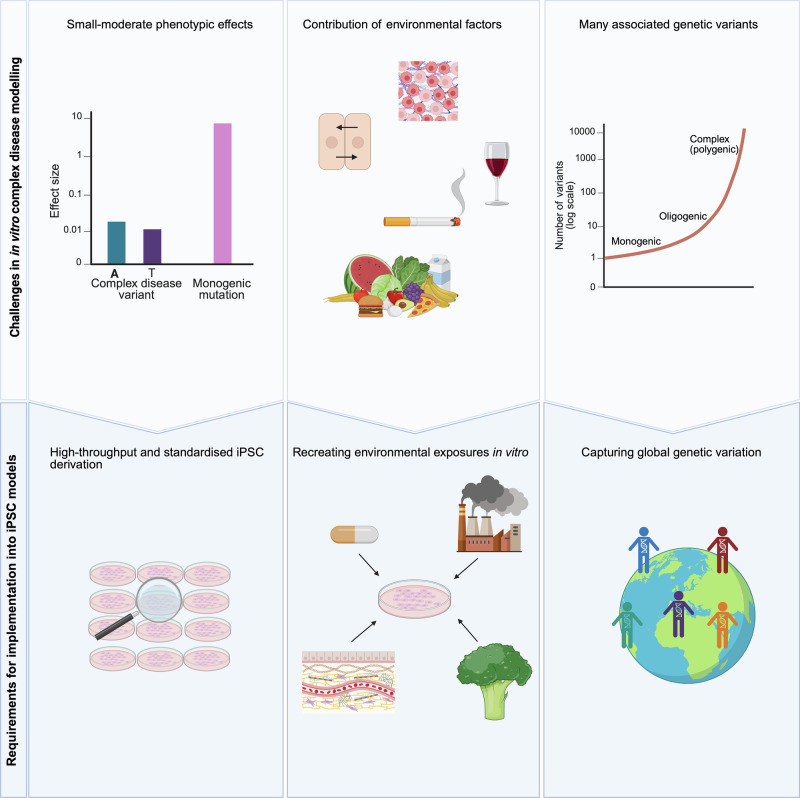
Challenges in iPSC-based complex disease modelling. Modelling complex diseases presents several unique challenges, including capturing subtle to moderate cellular effects, modelling environmental influences, and capturing the entire range of associated genetic variants. Various approaches are under development to meet the requirements for addressing these challenges, such as large-scale iPSC derivation, techniques to capture environmental factors, and iPSC biobanks to include more genetic diversity in iPSC models. iPSC induced pluripotent stem cell. Created with BioRender.

Modelling complex diseases introduces additional challenges. First, unlike monogenic disease variants with high penetrance, the genetic variants associated with complex diseases exert subtle to moderate phenotypic effects. Second, complex disease phenotypes are influenced by environmental factors that are difficult to capture in vitro due to their multifactorial nature. Finally, complex diseases are highly polygenic, and to capture the full spectrum of associated genetic variants, it is essential to incorporate global genetic diversity into the model (Fig. 2).

## Detecting subtle phenotypic effects: balancing scale and resolution

**Fig. 3:**
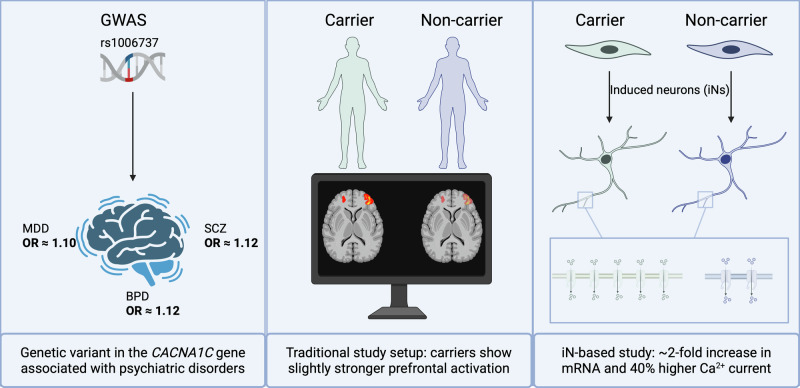
Effect sizes of complex disease variants across different biological scales. The effect size of complex disease genetic variants depends on the biological scale at which they are measured. On the molecular level, a SNP in the *CACNA1C* gene confers double the mRNA expression and a 40% higher Ca2+ influx, while the effect on the organismal level is substantially smaller, as measured by fMRI of the prefrontal cortex. SNP single nucleotide polymorphism, CACNA1C calcium voltage-gated channel subunit alpha 1, fMRI functional magnetic resonance imaging. Created with BioRender.

Monogenic disease variants typically confer highly penetrant phenotypic effects that are easily detectable in cellular systems, necessitating only few cell lines. For instance, iPSC-derived neurons carrying *SHANK3* haploinsufficiency (a monogenic cause of Phelan-McDermid syndrome) show striking cellular abnormalities, including reduced synapse formation, impaired dendritic arborization, and disrupted network activity [36]. In contrast, complex diseases are influenced by large numbers of genetic variants, each of which typically confers only a small effect on clinical outcomes, limiting their direct biological interpretability. In defined cellular contexts, these genetic variants may exhibit more pronounced molecular effects, although these remain substantially smaller than those related to monogenic mutations. For example, the psychiatric risk variant rs1006737 in the *CACNA1C* gene has a small clinical effect size (OR ≈ 1.1 [37, 38]) and has subtle effects observable on fMRI [39]. However, on the cellular level, induced neurons derived from homozygous carriers show substantial effects, with a nearly two-fold increase in *CACNA1C* mRNA expression and 40% higher calcium channel activity [40] (Fig. 3). This illustrates how cellular models can reveal mechanisms that remain difficult to resolve at the clinical level. Extending such studies to population-scale iPSC models would enable the simultaneous investigation of many genetic variants, allowing inter-individual genetic variation, including both GWAS loci and novel variants, to be statistically associated with cellular phenotypes in a manner conceptually analogous to eQTL mapping. Such approaches require sufficiently large sample sizes and rigorous control of technical variability to detect subtle effects.

Automated and semi-automated iPSC culturing systems have recently emerged to increase throughput and standardisation by performing tasks traditionally performed manually, such as reprogramming, clone selection, passaging and differentiation [33, 34, 41]. Automation is particularly advantageous for cell types requiring long differentiation times to achieve functional maturity, such as neurons. For instance, long-term co-cultures of neurons, astrocytes, and microglia have recapitulated Alzheimer’s disease phenotypes (amyloid-β plaque formation, neuritic dystrophy, synaptic loss) that are difficult to capture in manually maintained cultures, which are often short-term [41]. Although still developing, automated systems have the potential to combine standardisation with increased throughput, facilitating larger-scale, genetically informative iPSC studies that capture a broader spectrum of genetic effects on cellular phenotypes.

Another strategy to increase throughput involves pooled experimental paradigms (i.e. iPSC villages), in which lines from many donors are co-cultured in a single dish under uniform culture conditions [42]. The primary aim of such designs is to enable efficient discovery of genotype–phenotype relationships by reducing technical variability and increasing statistical power across many donors. In current paradigms, donor-specific molecular phenotypes are computationally resolved rather than analysed as bulk averages. Single-cell genetic demultiplexing assigns transcriptomic or proteomic profiles to individual donors within pooled cultures, enabling genotype–phenotype associations under shared conditions [42–45]. For instance, Census-seq has been used to quantify donor-specific protein expression in iPSC-derived neuronal villages [42], illustrating how genetic influences on cellular phenotypes can be resolved within pooled cultures. Nevertheless, the composition of iPSC villages should be tightly controlled, since lines can differ substantially in proliferation rates [43], resulting in competitive overgrowth. Moreover, inter-individual variation in differentiation efficiency has been observed, although inter-line signalling within the dish may improve this in some cell types [43], but not others [45]. While iPSC villages offer clear advantages for population-scale studies, the extent to which donor-specific molecular variation, particularly beyond transcriptional profiles, can be disentangled from inter-line interactions within the village remains to be determined.

## Beyond the genome: modelling environmental influences on complex diseases

Complex diseases arise from the interplay between genetic predispositions and environmental exposures [46]. Modelling environmental influences in cellular systems has proven challenging due to their dynamic and multifactorial nature. Cellular characteristics can be shaped by both the immediate microenvironment (e.g. extracellular matrix), and by broader macroenvironmental factors such as inflammation, toxin exposure, medication, or diet. Macroenvironmental factors vary widely between individuals in intensity, duration, and individual susceptibility, making these effects difficult to reproduce and standardise in experimental models.

The cellular microenvironment, comprising neighbouring cells, extracellular matrix (ECM), interstitial fluid, and physical forces (e.g. shear stress, stiffness gradients, perfusion) defines the local context in which complex disease mechanisms occur [47]. In the ideal experimental setting, ECM composition would be recapitulated, as well as dynamic processes such as fluid flow, nutrient and oxygen delivery, heterotypic cell-cell interactions (e.g. endothelial, immune, stromal) and mechanical competition for space. Current approaches partially address these requirements, where micropatterning of extracellular matrix substrates or cell-adhesion islands enables control over the cellular layouts [48]. Additionally, microfluidic organ-on-a-chip systems allow controlled perfusion, shear stress, and nutrition gradients in iPSC-derived cells, thereby more closely mirroring in vivo physiology [49, 50]. For example, a recent human heart-on-a-chip study incorporating iPSC-derived cardiomyocytes, endothelial cells and fibroblasts under flow showed alignment of endothelial cells and enhanced maturation of cardiomyocytes relative to static culture [51]. Nonetheless, most current iPSC systems typically model single environmental exposures in isolation rather than the complex, dynamic cellular context that maintains physiological tissue homeostasis [52]. While still evolving, these approaches can substantially improve the detection of gene–environment interactions in complex diseases.

Macroenvironmental factors shape cellular biology throughout life, influencing both early development and adult disease risk. In utero exposures, such as maternal stress and nutrition, establish epigenetic and metabolic patterns that can persist for decades [53, 54], often interacting with genetic predispositions to drive complex disease progression. At the population level, environment-wide association studies (EnWAS) have been developed to systematically assess a wide range of environmental exposures in relation to disease outcomes. For example, a seminal EnWAS of type 2 diabetes analysed associations between hundreds of environmental factors (e.g. pollutants and micronutrients) and diabetic status, identifying several exposure-disease links ranging from pesticide derivatives to vitamin intake [55]. Such studies have underscored the breadth of environmental factors that can be associated with diseases, and therefore also the difficulty of capturing such factors in modelling systems.

To date, most iPSC-based studies have focused on modelling single environmental exposures. For instance, cigarette smoke and its components have been studies in a range of iPSC-derived cell types under controlled in vitro conditions. iPSC-derived endothelial cells exposed to tobacco smoke increased oxidative stress, DNA damage/repair response, apoptosis and several other cellular pathways that associate with cellular stress [56]. Similarly, iPSC-derived cardiomyocytes have been exposed to smoke and nicotine-containing aerosol bubbled media and exhibited cardiotoxic metabolic changes [57]. Such work demonstrates the feasibility of using iPSC-based systems to interrogate the cellular consequences of defined macroenvironmental exposures. However, these approaches are inherently limited to environmental factors that can be directly measured and experimentally applied in vitro, and are less suited to capturing broader, cumulative, or indirectly acting exposures such as socioeconomic status, psychosocial stress, or complex lifestyle patterns.

Epigenetic mechanisms form a key interface between environmental exposures and the genome, translating environmental exposures to cellular responses such as DNA methylation and histone modifications [58]. Epigenome-wide association studies (EWAS) have linked a wide range of environmental factors to epigenetic changes across different developmental stages, from prenatal exposures such as folate levels [59], to broader factors including nutrition [60], medication [61], and socioeconomic status [62].

However, translating these associations into mechanistic insights within cellular systems remains challenging, as most EWAS are conducted using easily accessible cell types, such as blood cells, which provide limited insights for disease-relevant tissues. Recent advances in CRISPR Cas9 technology now enable targeted manipulation of DNA methylation at specific genomic loci, opening the door for analysing how environmentally induced epigenetic modifications affect cellular mechanisms [63]. Since some exposure-linked epigenetic markers are conserved across tissues (e.g. smoking [64]) and developmental stages (e.g. air pollution [65]), engineering these epigenetic markers in iPSC-derived cells may help identify how systemic environmental influences contribute to disease-relevant phenotypes.

## Enhancing genetic representation in iPSC models

Complex diseases are highly polygenic, involving thousands to potentially millions of genetic variants [66]. Capturing this full spectrum of genetic variation is essential to elucidate the molecular mechanisms underlying disease. However, most genetic studies have focused on individuals of European ancestry, representing only a fraction of global diversity. Expanding research to individuals beyond the European mainland is therefore critical to uncover ancestry-specific mechanisms that are rare or absent in European cohorts. Indeed, recent GWAS in African ancestry populations have identified unique genetic variants associated with various complex traits, such as BMI and Parkinson’s disease [67, 68]. Together, these findings underscore the importance of integrating genetic discovery with functional studies that are explicitly designed to capture genetic diversity, to ensure that inferred disease mechanisms are biologically and ancestrally representative. To this end, initiatives such as the iDA project [69] are developing ancestrally diverse iPSC panels to investigate how disease-associated genetic variants influence cellular mechanisms across populations.

Increasing genetic diversity in iPSC-based studies enhances the biological relevance of the model but also introduces additional sources of variation. Even within ancestrally homogeneous populations, inter-line variability can be substantial, arising from biological differences between donors and technical factors related to reprogramming and differentiation [26, 28]. Incorporating donors from multiple ancestral groups adds a further layer of ancestry-dependent differences in downstream biological mechanisms. These ancestry effects may covary with donor-level variation, complicating the delineation of variant-specific mechanisms from broader ancestry-related regulatory patterns. The resulting increase in heterogeneity can reduce statistical power, particularly in smaller cohorts, highlighting the need for balanced sampling strategies and analytical setups that explicitly model population structure when investigating cross-ancestry cellular phenotypes.

Ancestry-dependent variation has readily been observed during iPSC derivation, indicating that ancestral background has a substantial influence on pluripotency-associated genes [70]. Interestingly, these genes are enriched for pathways related to wound healing and cancer, suggesting that ancestry may influence cytoskeletal remodelling and protein localisation involved in the mesenchymal-to-epithelial transition, a critical step in acquiring pluripotency. Such insights underscore that even fundamental cellular processes may be ancestry-dependent, highlighting the importance of ancestrally diverse iPSC biobanks for both mechanistic discovery and the development of robust, generalisable disease models.

Emerging large-scale iPSC biobanks are marking a new era in complex disease modelling by providing access to thousands of genetically diverse cell lines. The California Institute for Regenerative Medicine (CIRM) iPSC repository currently includes more than 2500 iPSC lines derived from both healthy donors and patients of African, Asian, European, and Hispanic/Latino ancestries. These lines represent a wide range of complex diseases, including neurodegenerative, neurodevelopmental, cardiovascular and metabolic disorders, offering the opportunity to study cross-population disease mechanisms. Similarly, the WiCell repository is one of the most diverse iPSC biobanks, including ~40% of iPSC lines derived from African American, Arab, Asian, Latino, or Native American individuals. These and other initiatives set the stage for more comprehensive and translationally informative complex disease models.

Conventional GWAS begin with clinical outcomes and subsequently aim to uncover the biological mechanisms underlying associated loci. Population-scale iPSC studies enable the reverse approach by starting from experimentally tractable cellular phenotypes with measurable biological effects and trace these back to their genetic determinants [42, 71]. In such experiments, iPSCs or iPSC-derived cells are profiled for phenotypes of interest (e.g. cell morphology, proliferation) to identify variants that modulate cellular behaviour. This “functional-first” strategy directly links genetic variation to cellular mechanisms and yields variants with well-defined biological effects. These mechanisms can then be examined in clinical cohorts to assess their contribution to disease risk.

## Concluding remarks

Despite major progress in identifying genetic associations with complex diseases, translating these into functional mechanisms remains a key challenge. iPSC models provide a versatile framework to connect genetic variation to cellular phenotypes across multiple tissues and disease stages, but their reproducibility and scalability require further refinement. Advances in automation and pooled culture systems can provide the throughput needed for systematic, population-scale studies on complex disease mechanisms. Improving the physiological relevance of iPSC models will depend on better modelling of environmental influences and expanding genetic diversity within iPSC resources. As these developments converge, iPSC-based systems are poised to become a tool for bridging genetic risk and disease biology, offering functional resolution to human genomics.
